# Bifunctional role of some biogenic nanoparticles in controlling wilt disease and promoting growth of common bean

**DOI:** 10.1186/s13568-023-01546-7

**Published:** 2023-04-29

**Authors:** El-Sayed R. El-Sayed, Samar S. Mohamed, Shaimaa A. Mousa, Mohamed A. Abo El-Seoud, Adel A. Elmehlawy, Dalia A.M. Abdou

**Affiliations:** 1 Plant Research Department, Nuclear Research Center, Egyptian Atomic Energy Authority, Cairo, Egypt; 2 Microbiology Department, Faculty of Science, Ain Shams University, Cairo, Egypt

**Keywords:** Nanoparticles, *Fusarium oxysporum*, Plant pathogenic fungi, Wilt disease, *Phaseolus vulgaris* L., Sustainable agriculture

## Abstract

In the present era, nanomaterials are emerging as a powerful tool for management of plant disease and improving crop production to meet the growing global need for food. Thus, this paper was conducted to explore the effectiveness of five different types of nanoparticles (NPs) viz., Co_3_O_4_NPs, CuONPs, Fe_3_O_4_NPs, NiONPs, and ZnONPs as treatments for *Fusarium* wilt as well as their role in promoting growth of the common bean plant. The five types of NPs were applied as a treatment for wilt in two ways, therapeutic and protective plans under greenhouse conditions. In vivo experiments showed that all types of NPs significantly increased disease control and diminished the symptoms of *Fusarium* wilt for both incidence and severity. The recorded values for disease control using the respective NPs during the protective plan were 82.77, 60.17, 49.67, 38.23, and 70.59%. Meanwhile these values were 92.84, 64.67, 51.33, 45.61, 73.84% during the therapeutic plan. Moreover, CuONPs during the protective plan were the best among the five types of NPs employed in terms of wilt disease management. Regarding the use of these NPs as growth promoters, the obtained results confirmed the effectiveness of the five types of NPs in enhancing vegetative growth of the plant under greenhouse conditions, in comparison with control. Among the five NPs, CuONPs improved the plant vegetative growth and particularly increased the content of the photosynthetic pigments; chlorophyll-a (2.96 mg/g), -b (1.93 mg/g), and total carotenoids (1.16 mg/g). These findings suggest the successful and potential exploitation of nanomaterials in agriculture deployed as nano-based products including nano-fungicides and nano-fertilizers. In terms of sustainability, this promising and exceptional multifunctional role of these nanomaterials will surely exert positive impacts on both the environment and sustainable agriculture.

## Introduction

In the current scenario, there is an urgent scientific and social needs to meet the rapidly growing nutritional demands of the population (Lopez-Lima et al. 2021). Unfortunately, 20–40% of crops are lost each year due to microbial attacks, pest infestation, lesser nutrient availability, and poor soil quality (Worrall et al. 2018; Mittal et al. 2020). Among the phytopathogens, fungal infections cause about 70–80% of all the microbial diseases in agricultural systems (Kutawa et al. 2021; Peng et al. 2021). For example, wilt disease caused by *Fusarium oxysporum* (Jo et al. 2018) is a worldwide deadly vascular syndrome in several economically important crops such as lettuce and tomato (Lopez-Lima et al. 2021) due to its prolonged survival of the fungus in soil (Inami et al. 2014) and generation of resistant species to commercially available fungicides (Goffeau 2008). Fungal spores were found to survive for several years in a field (McGovern 2015). After infecting the plant parts, the fungus affects the water and nutrients flow in the plant (Arie 2010), resulting in yellowing, wilting, and plant death (Boix-Ruíz et al. 2015). Originally, the use of resistant cultivars and chemical fungicides can to some extent reduce the wilt disease. However, due to the variability in pathogenicity, the development of new pathogenic species is a continuing problem (McGovern 2015). In addition, the use of fungicides usually causes contamination problems of soil and plant tissues and they are not always effective and expensive (Sharma et al. 2018). For all of these reasons, developing a cost-effective and environmentally-friendly mitigation plans to overcome these issues by using more innovative technologies are immediately required.

Application of nanotechnology in agriculture has contributed to a revolution in the agrotechnology over recent years (Fiol et al. 2021) that completely changed the present farming practices. It led to the development of new concepts and agricultural products with immense potential to manage the aforementioned problems (Worrall et al. 2018) and has an imminent potential to reform the resilient agricultural system while promising food security (Mittal et al. 2020). Thus, disease management in plants using nanotools could be useful in providing protection to plants against pathogens (Sathiyabama 2019) or disease management through highly regulated and targeted disposition of active ingredients at required sites (Zhera et al. 2021). Several nanoparticles (NPs) and nanoparticle-based formulations such as nano-fungicides (Fu et al. 2020), nano-fertilizers (Banerjee et al. 2021), nano-pesticides, and nano-herbicides have been widely used for both soil improvement and plant health management (Khan et al. 2021). In general, the use of fungal cultures for the synthesis of NPs has magnificently emerged as the most advantageous (El-Sayed et al. 2023). Over the years, fungi have been employed extensively in several industrial applications (El-Sayed 2021; El-Sayed et al. 2020a; b; c; El-Sayed and Zaki 2023) as the most efficient biotechnological factories due to their flexibility, simplicity, easy maintenance, and tolerance (El-Sayed et al. 2022a; b; c). In this regard, the use of endophytes has emerged as a new research area for green and cost-effective production of several NPs (Abdelhakim et al. 2020; El-Sayed et al. 2022a; Hussein et al. 2022). This novel research area will open a new horizon, on the interface of nanotechnology and microbiology, for novel synthesis of diverse nanomaterials with several applications (Anwar et al. 2022; Hatab et al. 2022;).

In this regard, we have already described the synthesis of Co_3_O_4_NPs, CuONPs, Fe_3_O_4_NPs, NiONPs, and ZnONPs using the endophytic fungus *Aspergillus terreus* (Mousa et al. 2021). All the five NPs showed promising in vitro antifungal potential against several plant and human pathogens. However, few studies in the literature focused on evaluating these types of NPs against *Fusarium* wilt causing pathogens (Lopez-Lima et al. 2021). This encouraged us to explore the in vivo antifungal potential of the five types of NPs against *Fusarium* wilt disease in *Phaseolus vulgaris* L. plant under greenhouse conditions. *Phaseolus vulgaris L*. is an extremely important aliment, (Rivera et al. 2018) not only representing the main source of dietary protein for humans in several world regions (but also contributing greatly to diet with starch, fiber, vitamins, and minerals (Hayat et al. 2014). Further, the effectiveness of the five types of NPs in promoting growth of the *P. vulgaris* plant was also investigated in this study.

## Materials and methods

### Plant and soil

Experimental research and field studies on plants (either cultivated or wild), including the collection of plant material, were performed in this study according to the institutional, national, and international guidelines and legislation. This study was carried out at the experimental plantation area of the Nuclear Research Center, Egyptian Atomic Energy Authority during Spring 2020. Common bean (*Phaseolus vulgaris* L.) seeds were obtained from the Agronomy Institute, Agricultural Research Centre, Giza, Egypt (http://www.arc.sci.eg/). The soil used in this study was a mixture of peat moss (PINDSTRUP MOSEBRUG A/S, Latvia) and sandy soil (1:1, w/w). The soil mixture was sterilized and distributed in pots. The sandy soil was subjected to analyses (Carter and Gregorich 2008) and all the mechanical, physical, and chemical parameters were listed in Table 1.

**Table 1:** Mechanical, physical, and chemical properties of sand soil used in the present study

Mechanical properties			Physical properties			Chemical properties	
Coarse sand	29.50%		Saturation	21.45%		Organic matter	0.31 g kg^− 1^
Fine sand	68.50%		EC (ds m^− 1^)	3.13		Available N	5.02 (mg kg^− 1^)
Clay	0.0%		Available water	10.02%		Available P	2.11 (mg kg^− 1^)
Silt	2.0%		Bulk density	1.64 (g cm^− 3^)		Available K	0.23 (mg kg^− 1^)
Texture	Sandy		pH (1: 2.5)	7.25		CaCO_3_	0.00 g kg^− 1^

### Nanoparticles

Co_3_O_4_NPs, CuONPs, Fe_3_O_4_NPs, NiONPs, and ZnONPs prepared during our previous studies (Mousa et al. 2021; El-Sayed et al. 2022c) were used in the present study. All the NPs were characterized using several techniques including transmission electron microscope, X-ray diffraction, dynamic light scattering analysis, UV–Vis, and Fourier transform infrared spectroscopy. The mean particle sizes of the respective NPs were 10.35, 18.95, 32.41, 42.51, and 30.45 nm (Mousa et al. 2021).

### Evaluation of the efficacy of NPs against wilt disease

#### Preparation of greenhouse experiments

Seeds were disinfected by immersion in 70% ethanol for 2 min then washed with sterile distilled water several times and let to dry. Seeds were sown at 3 cm depth in a 30 cm plastic pots filled with the prepared soil. Pots were kept under natural light and temperature conditions and were irrigated regularly every 3 days with equal amounts of sterilized distilled water. Each treatment was replicated 3 times with ten plants/replicate.

#### Induction of Fusarium wilt disease and treatment of plants

The plant pathogen, *Fusarium oxysporum* EUM37 was obtained from the Microbiological Resources Centre (Cairo MIRCEN), Faculty of Agriculture, Ain Shams University, Cairo, Egypt. The strain was cultivated in PD broth medium for 5 days at 30℃. The obtained spore was immersed in sterile distilled water in a concentration of 10^6^ spore/mL. 25 mL of this suspension was applied to each pot to induce the disease. Sterile distilled water (25 mL) served as the uninoculated positive control.

Mitigation of the wilt disease using Co_3_O_4_NPs, CuONPs, Fe_3_O_4_NPs, NiONPs, and ZnONPs was evaluated in two ways. The first is a therapeutic plan in which the plant was infected and then treated with NPs after germination. The other is a protective plan which consist of treating the plant with NPs from the first day before infection. NPs were dispersed in aqueous suspension at a concentration of 100 mg L^− 1^ (Co_3_O_4_NPs and CuONPs), 250 mg L^− 1^ (Fe_3_O_4_NPs and NiONPs), and 500 mg L^− 1^ (ZnONPs), the minimum inhibitory concentrations against *F. oxysporum*, according to our previous study (Mousa et al. 2021). All the dispersed NPs were treated ultrasonically before use and added to the irrigation water. Full description of the employed treatments was listed in Table 2.

**Table 2:** **Treatment plans, symbols, and their description used against** ***Fusarium*** **wilt disease**

Treatment	Symbol	Description
Positive control	T1	Control plant
Negative control	T2	Infected plant without treatment
Therapeutic plan	T3	Plant treated with CuONPs after infection
	T4	Plant treated with Co_3_O_4_NPs after infection
	T5	Plant treated with Fe_3_O_4_NPs after infection
	T6	Plant treated with NiONPs after infection
	T7	Plant treated with ZnONPs after infection
Protective plan	T8	Plant treated with CuONPs before infection
	T9	Plant treated with Co_3_O_4_NPs before infection
	T10	Plant treated with Fe_3_O_4_NPs before infection
	T11	Plant treated with NiONPs before infection
	T12	Plant treated with ZnONPs before infection

#### Disease assessment

After 60 days of inoculating the pathogen, the wilt disease was evaluated. To determine the disease incidence, the leaves with symptoms and the total leaves in each plant were recorded.

Disease incidence (%) = (Number of diseased leaves / Number of total leaves) × 100.

Disease control (%) = 100 – % of Disease incidence.

To determine the disease severity, all the diseased leaves were visually inspected, which determined the percentage of the surface with symptoms of necrosis and yellowing.

Disease severity (%) = (average surface area of diseased leaves / total surface area of diseased leaves) × 100. Moreover, plant height (cm), root length (cm), fresh and dry weights (g/plant), and number of leaves/plant were reorder for each plant.

### Mode of action of CuONPs against Fusarium wilt pathogen

The antifungal action of CuONPs on the morphology of *F. oxysporum* mycelium was studied by Scanning Electron Microscopy (SEM) according to the protocol of Pochon et al. (2012) with some modifications. In 50 mL of PD broth, spore suspension of *F. oxysporum* was inoculated and allowed to grow for three days in static conditions. After 3 days of inoculation, the flasks were supplemented with of CuONPs (100 µg mL^− 1^) in different flasks for 48 h. Fungal mycelia grown were filtered and fixed under vacuum using 6% glutaraldehyde at room temperature for 24 h, rinsed three times (with 0.02 M phosphate buffers). Subsequently, it was fixed for 24 at 20 °C with 2% osmium tetra oxide, dehydrated (in a graded ethanol series for five min), and dried by CO_2_ (SAMDRI 780-B Tousimis). Finally, the sputter coated with gold palladium in a Nanotech sputter coater (BAL-TEC SDC 050). The prepared samples were examined with a Quanta 250 FEG scanning electron microscope operated at 30 kV.

### Effect of Co_3_O_4_, CuO, Fe_3_O_4_, NiO, and ZnO NPs on the growth of ***P. vulgaris*** plants

In this experiment, role of the five types of NPs as growth promoters were individually evaluated. NPs were dispersed in aqueous suspension at the same concentration (100 mg L^− 1^) and added to the irrigation water. Plants were treated three times, at planting of seeds, then two times every 15 days.

To evaluate the role of NPs as growth promoters, the following data for each NPs treatment were recorded, germination percentage, plant height (cm), root length (cm), fresh and dry weights (g/plant), and number of leaves/plant. In addition, Chlorophyll a, b, total chlorophyl, and total carotenoid concentrations were spectrophotometrically determined according to the method descried by Gross (1991). In brief, 100 mg fresh leaf materials were ground in a pre-chilled mortar in the presence of 8 mL of 80% acetone. The mixture was filtered and the volume adjusted to 10 mL with cold acetone. Finally, the absorbance of the extract was read at 645 nm (chlorophyll-a), 663 nm (chlorophyl-b), 672 (total chlorophyl) nm, and 470 nm (total carotenoids).

### Statistics

Statistical significance of the obtained means was analyzed by the analysis of variance (One-Way ANOVA) and the Least Significant Difference (LSD) tests (at 0.05 level) using SPSS software version 22 (IBM Corp).

## Results

### Biocontrol of Fusarium wilt disease by Co_3_O_4_NPs, CuONPs, Fe_3_O_4_NPs, NiONPs, and ZnONPs

**Fig. 1:**
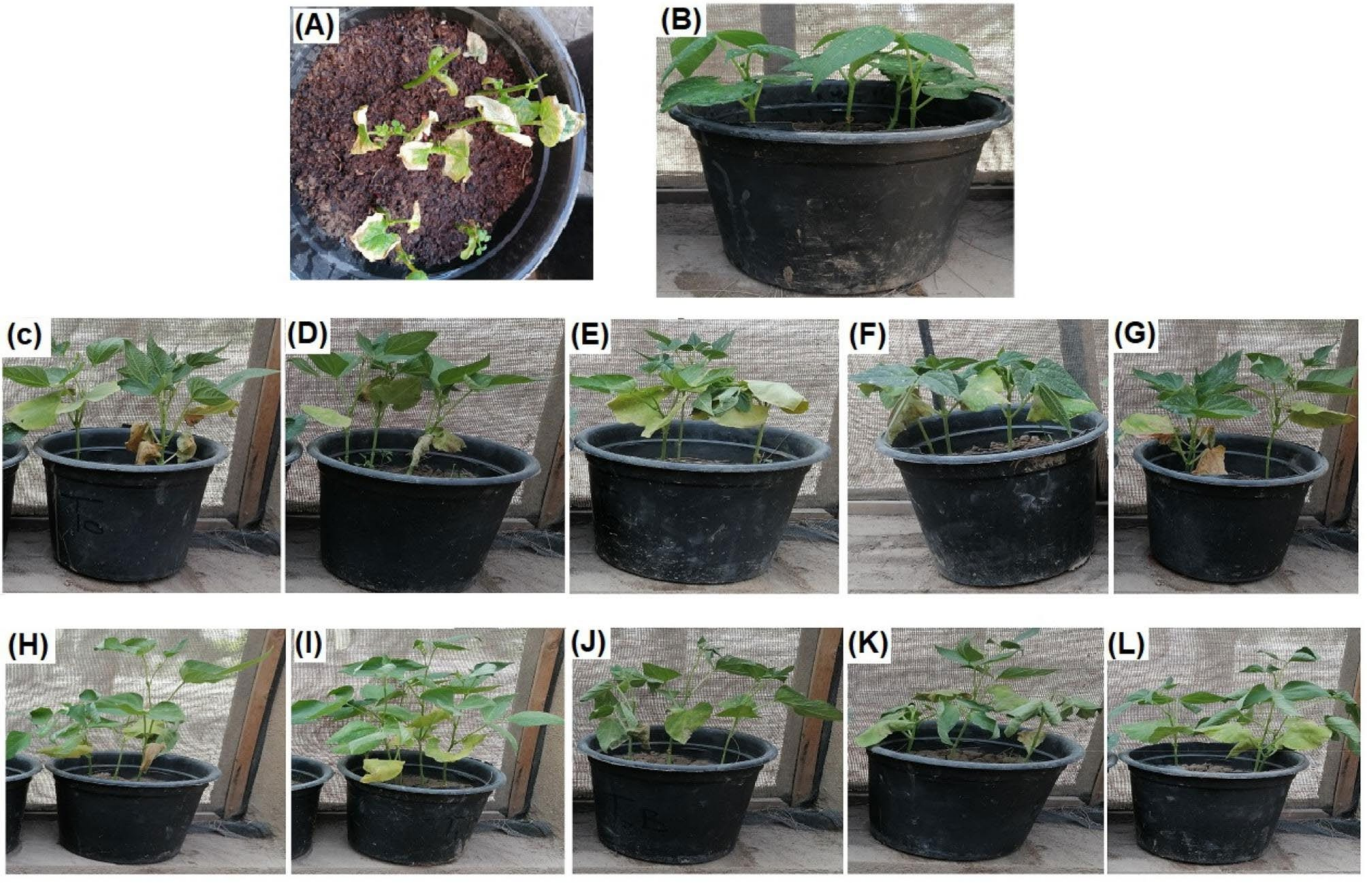
Photographs of the *Phaseolus vulgaris* plant controls; Infected un-treated **(A)**, Un-infected **(B)**, the therapeutic plan; Treatment with Co_3_O_4_NPs **(C)**, Treatment with CuONPs **(D)**, Treatment with Fe_3_O_4_NPs **(E)**, Treatment with NiONPs **(F)**, and Treatment with ZnONPs **(G)**, and the protective plan; Treatment with Co_3_O_4_NPs **(H)**, Treatment with CuONPs **(I)**, Treatment with Fe_3_O_4_NPs **(J)**, Treatment with NiONPs **(K)**, and Treatment with ZnONPs **(L)**

The disease severity, incidence, and control were recorded against the wilt disease by using the five types of biosynthesized NPs at two mitigation plans. Table 3 presented the recorded values of the severity, incidence, and control of wilt disease. A noticeable reduction was observed in the agro-morphological attributes of the plants infected with the *Fusarium oxysporum* (Fig. 1A). Meanwhile, it was observed that the five biosynthesized NPs inhibited the wilt disease progression (Fig. 1C-L). Result in Table 3 showed that the applied NPs had different and varied impacts on the studied parameters for both the therapeutic and protective experiments. It is obvious that the CuONPs was the most effective treatment to decline the negative effect of *Fusarium* wilt disease on the plant followed by ZnONPs, Co_3_O_4_NPs, Fe_3_O_4_NPs, and NiONPs. That holds true for both the therapeutic (Fig. 1C-G) and protective (Fig. 1H-L) plans. Comparing the influence of the NPs under investigation for either the therapeutic or the protective experiments, it could be easily deduced that the impact of the studied NPs was much more promising in the protective plan rather than therapeutic plan.

**Table 3:** Disease severity, incidence, and control of different treatment plans against wilt disease

Treatments		Disease severity (%)	Disease incidence (%)	Disease control (%)
Control	T1	0.00^ h^	0.00^i^	100
Negative control	T2	98.35 ± 8.66^a^	91.5 ± 5.33^a^	8.53
Therapeutic plan	T3	25.87 ± 5.25^f^	17.23 ± 2.65^ g^	82.77
	T4	44.63 ± 4.54^e^	39.83 ± 2.88^e^	60.17
	T5	63.56 ± 7.24^c^	50.33 ± 3.53^ cd^	49.67
	T6	72.54 ± 4.66^b^	61.77 ± 2.75^b^	38.23
	T7	39.55 ± 1.76^e^	29.41 ± 3.88	70.59
Protective plan	T8	18.76 ± 2.05^ g^	7.16 ± 0.76^ h^	92.84
	T9	40.78 ± 4.56^e^	35.33 ± 5.68^e^	64.67
	T10	55.87 ± 3.78^d^	48.67 ± 3.47^d^	51.33
	T11	68.45 ± 4.66^c^	54.39 ± 2.68^c^	45.61
	T12	41.67 ± 1.21^e^	26.16 ± 3.94^f^	73.84

Calculated mean is for triplicate measurements from two independent experiments ± SD, ^a−i^ means with different superscripts in the same column for each nanoparticle are considered statistically different (LSD test, *P* ≤ 0.05)

**Fig. 2:**
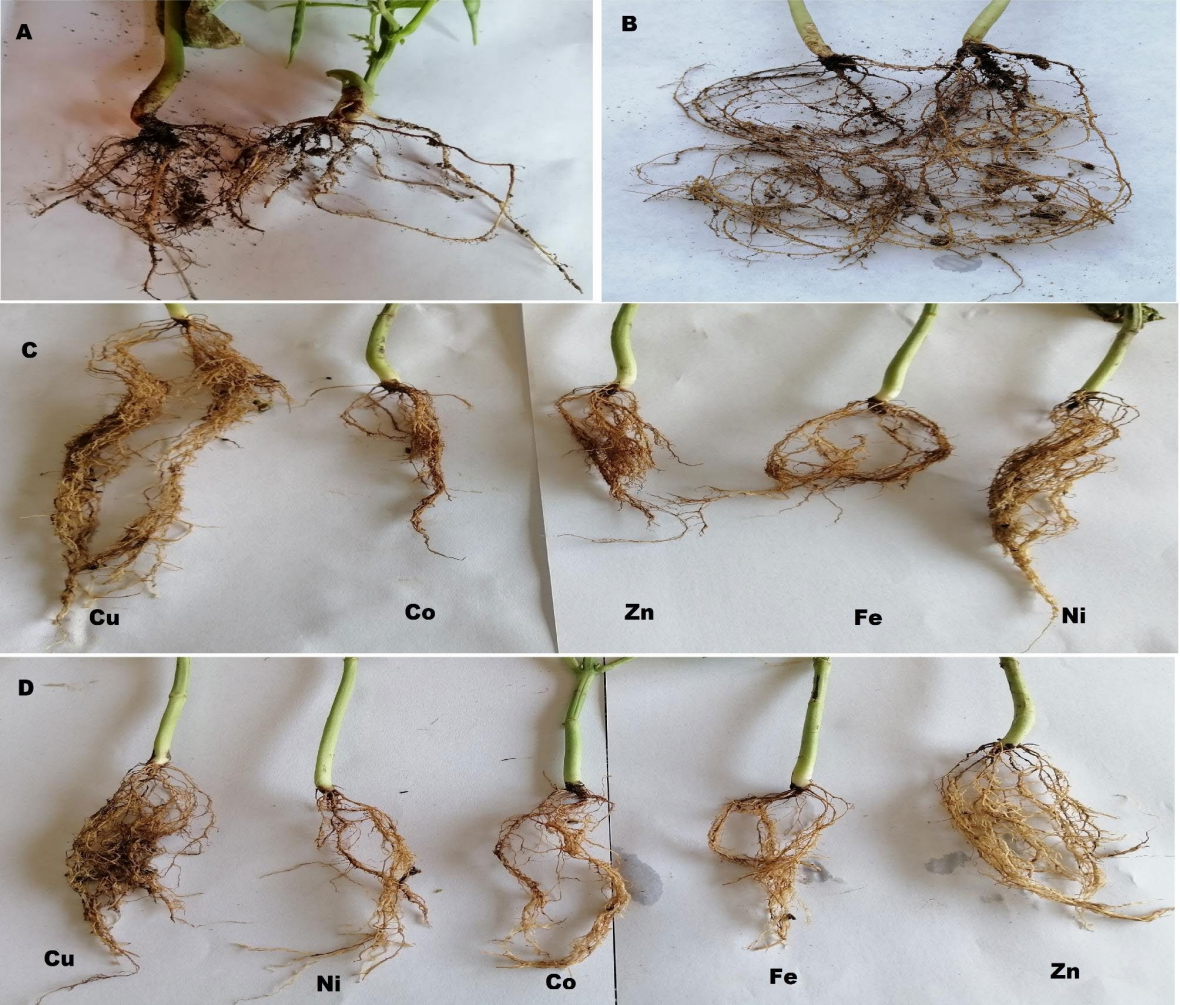
Photographs of roots of *Phaseolus vulgaris*. Roots of infected plants **(A)**, Roots of control plants **(B)**, Roots of infected plants reated with NPs according to the protective plan **(C)**, and Roots of infected plants reated with NPs according to the protective plan **(D)**

Data recorded in Table 4 presented the results of vegetative growth parameters of different treatment plans against wilt disease. Naturally, infecting red bean plants with the *Fusarium* wilt disease (Fig. 1A) was accompanied with a decrease in all the vegetative growth parameters. However, treating plants either before or after the infection with the NPs (Fig. 2) led to an increase and improvement in the above-mentioned growth parameters. In this connection, all the applied NPs raised the values of the vegetative growth parameters as compared with control. The most superior NPs to maintain plant growth was CuONPs followed by ZnONPs. More or less the same trend was observed for both the therapeutic and protective plans. However, the values of the protective plan showed slight increase more than the therapeutic plan.

**Table 4:** Measured vegetative growth parameters of different treatment plans against wilt disease

Treatments		Plant height (cm)	No of leaves per plant	Root length (cm)	Fresh weight (g/plant)	Dry weight (g/plant)
Control	T1	21.65 ± 0.80^a^	11.55 ± 0.19^a^	11.00 ± 0.50^a^	7.46 ± 0.15^a^	0.82 ± 0.01^a^
Negative control	T2	10.57 ± 0.50^c^	4.77 ± 0.50^c^	5.00 ± 0.40^c^	2.32 ± 0.07^c^	0.18 ± 0.02^b^
Therapeutic plan	T3	19.82 ± 0.15^a^	7.99 ± 0.01^a^	9.33 ± 0.52^a^	5.38 ± 0.15^bc^	0.70 ± 0.02^a^
	T4	16.33 ± 0.57^b^	6.41 ± 0.60^bc^	7.33 ± 0.57^bc^	4.88 ± 0.10^bc^	0.60 ± 0.01^a^
	T5	16.21 ± 0.57^b^	6.25 ± 0.60^bc^	6.01 ± 0.33^c^	4.77 ± 0.15^bc^	0.68 ± 0.01^a^
	T6	16.01 ± 0.33^b^	6.21 ± 0.65^bc^	6.88 ± 0.62^c^	4.98 ± 0.05^bc^	0.69 ± 0.01^a^
	T7	18.01 ± 0.66^a^	7.89 ± 0.01^a^	6.89 ± 0.11^c^	5.23 ± 0.05^bc^	0.67 ± 0.04^a^
Protective plan	T8	20.50 ± 0.33^a^	9.11 ± 0.90^a^	10.60 ± 0.10^a^	6.89 ± 0.01^b^	0.77 ± 0.05^a^
	T9	16.77 ± 0.50^b^	7.79 ± 0.10^a^	8.82 ± 0.20^b^	5.57 ± 0.08^bc^	0.70 ± 0.08^a^
	T10	16.89 ± 0.80^b^	7.86 ± 0.55^a^	7.02 ± 0.01^bc^	4.42 ± 0.08^bc^	0.73 ± 0.04^a^
	T11	16.64 ± 0.10^b^	7.66 ± 0.21^a^	9.49 ± 0.05^a^	4.26 ± 0.03^bc^	0.69 ± 0.01^a^
	T12	19.05 ± 0.58^a^	8.93 ± 0.33^a^	8.33 ± 0.80^b^	5.76 ± 0.05^bc^	0.65 ± 0.07^a^

Calculated mean is for triplicate measurements from two independent experiments ± SD, ^a−c^ means with different superscripts in the same column for each nanoparticle are considered statistically different (LSD test, *P* ≤ 0.05)

### SEM study of the effect of CuONPs on ***Fusarium oxysporum***

**Fig. 3:**
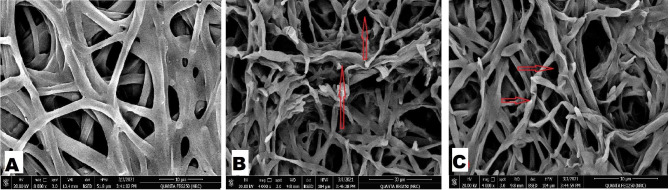
SEM micrographs of *Fusarium oxysporum.***(A)** Control (without CuONPs), **(B to F)** Dehydrated and distorted CuONPs treated mycelium

Figure 3 presented morphology of *F. oxysporum* mycelia after treatment with CuONPs as well as control treatments. In the case of control samples, the mycelia showed a clear germination and smooth surface (Fig. 3A). In contrast, after treatment with CuONPs resulted in a rougher surface of the mycelia with shrinking (Fig. 3B and C). In addition, the mycelium was distorted with intense dehydration that resulted in completely damaged or dead cells.

### Effect of Co_3_O_4_NPs, CuONPs, Fe_3_O_4_NPs, NiONPs, and ZnONPs on the development of ***P. vulgaris*** plant

**Fig. 4:**
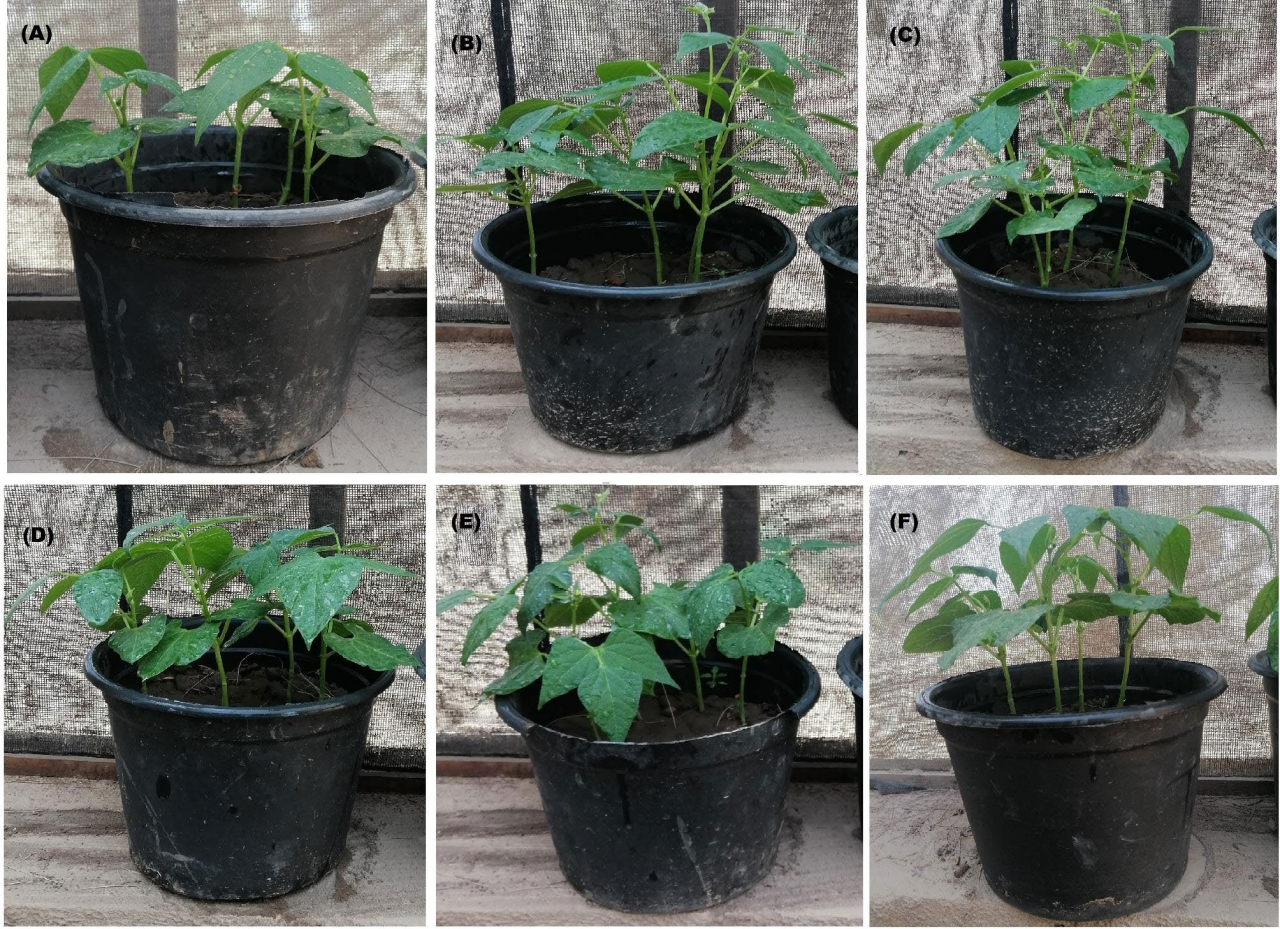
Photographs of the effect of different NPs on promoting growth of *Phaseolus vulgaris*. Treatment with Co_3_O_4_NPs **(A)**, Treatment with CuONPs **(B)**, Treatment with Fe_3_O_4_NPs **(C)**, Treatment with NiONPs **(D)**, and Treatment with ZnONPs **(E)**

Results presented in Table 5 confirmed the promotion effect of the applied NPs on germination percent and vegetative growth of *P. vulgaris* plant. Generally, the applied NPs increased the germination percent as compared to the control. CuONPs raised the germination percent to 100% in comparison with 76.66% for the control. The next values of germination percent were 94.12% for ZnONPs, 93.51% for Co_3_O_4_NPs, 86.83% for Fe_3_O_4_NPs, and 82.16% for NiONPs. Regarding the other vegetative growth parameters, it could be deduced that all the applied NPs significantly promoted the plant growth as it showed much more values rather than those of control (Fig. 4). The much more promoting NPs were CuONPs followed by Co_3_O_4_NPs, ZnONPs, NiONPs and Fe_3_O_4_NPs.

**Table 5:** Impact of NPs on the germination and vegetative growth parameters of *P. vulgaris*

Treatments	Germination (%)	Plant height (cm)	No of leaves per plant	Root length (cm)	Fresh weight (g/plant)	Dry weight (g/plant)
Control	75.06 ± 0.11^d^	21.43 ± 0.72^e^	11.55 ± 0.19^b^	11.11 ± 0.33^b^	7.51 ± 0.32^b^	0.84 ± 0.22^b^
CuONPs	100 ± 0.00^a^	36.00 ± 0.31^a^	17.00 ± 0.33^a^	16.00 ± 0.33^a^	13.53 ± 0.15^a^	2.06 ± 0.03^a^
Co_3_O_4_NPs	93.51 ± 5.07^b^	24.33 ± 0.15^d^	14.00 ± 0.00^ab^	12.33 ± 0.57^a^	8.30 ± 0.17^ab^	1.21 ± 0.03^a^
Fe_3_O_4_NPs	86.83 ± 2.88^c^	27.00 ± 0.33^c^	15.33 ± 0.67^ab^	13.66 ± 0.57^a^	9.18 ± 0.19^ab^	1.57 ± 0.06^a^
NiONPs	82.16 ± 7.66^c^	24.66 ± 0.55^d^	14.00 ± 0.33^ab^	12.66 ± 0.57^a^	8.70 ± 0.26^b^	1.17 ± 0.09^a^
ZnONPs	94.12 ± 3.76^b^	34.00 ± 0.24^b^	16.50 ± 0.50^a^	15.00 ± 0.28^a^	10.33 ± 0.10^ab^	1.89 ± 0.05^a^

Calculated mean is for triplicate measurements from two independent experiments ± SD, ^a−e^ means with different superscripts in the same column for each nanoparticle are considered statistically different (LSD test, *P* ≤ 0.05)

Data in Table 6 explained the impact of the applied NPs on pigment content of red bean. Data clearly showed that all applied NPs had the capacity to significantly increase leaves pigment content. The most superior NPs to promote pigment synthesis were CuONPs. That holds true for chlorophyll a, chlorophyll b, and consequently total chlorophyll. The same trend was concluded for carotenoids content. Maximum pigments values were exerted by CuONPs as it were 2.96, 1.93, 5.21 and 1.16 mg/g for chlorophyll a, chlorophyll b, total chlorophyll and carotenoids content. Thus, the promising impact of NPs on pigments synthesis was assured here in in comparison with the control.

**Table 6:** Impact of NPs on chlorophyl-a, chlorophyll-b, total chlorophyll, and total carotenoids of *P. vulgaris*

Treatments	Chlorophyll-a (mg/g)	Chlorophyll-b (mg/g)	Total chlorophyll (mg/g)	Total carotenoids (mg/g)
Control	2.08 ± 0.03^c^	1.02 ± 0.01^c^	3.14 ± 0.04^e^	0.55 ± 0.02^b^
CuONPs	2.96 ± 0.01^a^	1.93 ± 0.02^a^	5.21 ± 0.11^a^	1.16 ± 0.21^a^
Co_3_O_4_NPs	2.25 ± 0.05^b^	1.17 ± 0.01^b^	4.21 ± 0.05^c^	1.06 ± 0.11^a^
Fe_3_O_4_NPs	2.13 ± 0.01^b^	1.23 ± 0.06^b^	3.76 ± 0.06^d^	1.00 ± 0.00^a^
NiONPs	2.16 ± 0.06^b^	1.48 ± 0.07^b^	3.89 ± 0.03^d^	1.01 ± 0.32^a^
ZnONPs	2.85 ± 0.02^a^	1.79 ± 0.04^a^	4.82 ± 0.10^b^	1.13 ± 0.13^a^

Calculated mean is for triplicate measurements from two independent experiments ± SD, ^a−c^ means with different superscripts in the same column for each nanoparticle are considered statistically different (LSD test, *P* ≤ 0.05)

## Discussion

Till now, the use of nanotechnology in agriculture, in particular for plant protection and management of disease, is an under-explored area in the research community. Thus, the presented research was conducted to valorize the effect of five NPs as nanofungicides against *F. oxysporum* the common cause of wilt disease in *P. vulgaris*. Here, the role of Co_3_O_4_, CuO, Fe_3_O_4_, NiO, and ZnO NPs on controlling *Fusarium* wilt disease of *P. vulgaris* L. was evaluated in two ways. The first is a therapeutic plan in which the plant was infected and then treated with NPs after germination. The second is a protective plan, which consists of treating the plant with NPs from the first day before infection. Our results revealed that all the five NPs were effective in reducing the wilt disease, and suppression the disease severity when compared to infected plants. Among the five tested NPs, CuONPs was the most effective and significantly reduced *Fusarium* wilt disease in both the used mitigation plans. The highest control of wilt disease, lowest values of disease incidence and severity were obtained with the application of CuONPs in the protective plan. In addition, the application of CuONPs using the protective plan increased the vegetative growth parameters as compared to control plant. In agreement with our results, Ashraf et al. (2021) concluded that application of CuONPs to infected tomato by *F. oxysporum* significantly reduced the disease and improved the plant growth indexes and fruit quality. Elmer et al. (2021a) reported that application of CuONPs suppresses *Fusarium* wilt development on *Chrysanthemum*. In the literature, CuONPs were superior to other NPs in reducing disease symptoms for other crops, including eggplant (Elmer et al. 2021b), soybeans (Ma et al. 2020; Peréz et al. 2020), tomato (Ma et al. 2019), watermelon (Borgatta et al. 2018; Elmer et al. 2018), tea plants (Ponmurugan et al. 2016), and wheat (El-Sharkawy and El-Shora 2020). Generally, Copper is a cofactor for three proteins (plastocyanins, peroxidase and multi-Copper oxidase) which support the creation of the host defense barriers (Elmer et al. 2018). As such, poly phenol oxidase is enzyme of the major group that show increased activity in the presence of copper ions when plant attacked by pathogen. Our results further showed that ZnONPs reduced *Fusarium* wilt disease in both treatments. In recent study, Abdelaziz et al. (2021) studied the protective role of ZnONPs-based hydrogel against wilt disease caused by *F. oxysporum* of pepper plant. Our results also concluded the positive role of Co_3_O_4_, Fe_3_O_4_, and NiO, NPs against *Fusarium* wilt disease. This is the first study on the in vivo application of Co_3_O_4_NPs in plant disease management. All previous studies used Co_3_O_4_NPs as a nano-fertilizer (Jahani et al. 2019, 2020; Alam et al. 2019) studied the antibacterial efficacy of Fe_3_O_4_NPs against bacterial wilt pathogen *(Ralstonia solanacearum*). Also, Cai et al. (2020) evaluated the effect of Fe_3_O_4_NPs on *Nicotiana benthamiana* and plant resistance response against *Tobacco mosaic virus*. Ahmed et al. (2016) used NiNPs for controlling *Fusarium* wilt on lettuce and tomato. Further Sharma et al. (2017) used nickel ferrite NPs as fungicides against phytopathogenic fungi, *Colletotrichum gloeosporioides* and *F. oxysporum.*

In this study, scanning electron microscopy used to explore the antifungal effect of CuONPs as the most promising fungicide against *F. oxysporum*. The data of SEM in the present study showed that the fungal cell wall remained intact and the hyphae showed the normal structural property of *F. oxysporum* in the absence of CuONPs. In contrast, the cell wall of *F. oxysporum* was severely damaged and the hyphae had abnormal structural property in the presence of CuONPs. To the best of our knowledge, literature studies revealing the ivivo antifungal effect of CuONPs on *Fusarium oxysporum* are rare. Ashraf et al. (2021) studied the inhibition mechanism of green-synthesized CuONPs from *Cassia fistula* towards *Fusarium oxysporum* by boosting growth and defense response in tomatoes. The authors reported the same observations in agreement with our results. Moreover, similar observations were reported by Xia et al. (2016) and Ibrahem et al. (2021) with different types of NPs.

In addition to their role as a fungicide for controlling pathogens, the five types of NPs were also used as a growth promoting agents in this study. Our results indicated the stimulatory effect of all the applied NPs on germination percent, vegetative growth parameters, and photosynthetic pigments of *P. vulgaris* plant. The much more stimulating NPs were found to be CuONPs. In the literature, Da Costa and Sharma (2016) reported that CuONPs increased level of oxidative and osmotic stress, higher expression of ascorbate peroxidase and superoxide dismutase enzymes of rice. Also, CuONPs were used to enhance the growth rate and germination of several plants (Kasana et al. 2017; López-Vargas et al. 2018; Jahani et al. 2020) reported the positive effects of Co_3_O_4_NPs on ion leakage, total phenol and antioxidant enzymes activities of *Brassica napus* L. Also, Jahani et al. (2019) studied the effects of foliar application of Co_3_O_4_NPs on some physiological and biochemical changes of canola (*Brassica napus* L.) leaves. The previous study documented that the foliar spray of ZnONPs in *Coffea arabica* enhanced the net photosynthesis rate and biomass accumulation (Rossi et al. 2019). ZnONPs stimulated seed vigor, germination, early flowering, enhanced root and shoot growth (Prasad et al. 2012), enhanced photosynthetic pigments, and nutritive values of seeds (Patra et al. 2016). Generally, several studies reported the positive role of ZnONPs on plant physiology and growth parameters such as eggplant (Khan and Siddiqui 2018), *Triticum aestivum* (Awasthi et al. 2017), and *Leucaena leucocephala* (Venkatachalam et al. 2017). Regarding the use of Fe_2_O_3_NPs, Ibrahem et al. (2021) investigated the impact of different crystal sizes of Fe_2_O_3_NPs on wheat, foliar application of small particle size of iron oxide NPs induced photosynthetic pigments and total phenolic compounds contents. Similar to Elizabeth et al. (2020) that revealed the stimulation effect of Fe_2_O_3_NPs on tomato seeds and their effects on growth. There are some studies used the Fe_2_O_3_NPs as nanofertilizer and plants growth promoter in different plants such as peanut (Rui et al. 2016); spinach (Jeyasubramanian et al. 2016); *Nicotiana benthamiana* (Cai et al. 2020) and chickpea (Irum et al. 2020). Genreally, nanofertilizers offer a unique possibility to develop into plant nutrients that have high absorption rate and greater utilization efficacy and have minimum losses that facilitates the nutrients uptake to the plants (Fatima et al. 2021).

In summary, controlling the *Fusarium* wilt disease in *Phaseolus vulgaris* L. was carried out by five NPs. The applied NPs had different and varied impacts on the studied parameters for both the therapeutic and protective mitigation plans. It could be easily deduced that the impact of the studied NPs was much more promising in the protective plan rather than the therapeutic one. It is obvious that the CuONPs was the most effective treatment to decline the negative effect of *Fusarium* wilt disease followed by ZnONPs, Co_3_O_4_NPs, Fe_3_O_4_NPs and NiONPs. In addition to the role of the NPs as a fungicide for controlling pathogens infected growing plant, their role as a promoting agent was also considered. The data revealed that the promotion effect of the applied NPs on germination percent and vegetative growth of *P. vulgaris* plant.

## Data Availability

The authors confirm that the data supporting the findings of this study are available within the article.
